# Use of reprocessed external fixators in orthopaedic surgery: a survey of 243 orthopaedic trauma surgeons

**DOI:** 10.1186/s13037-018-0156-2

**Published:** 2018-06-07

**Authors:** Sorawut Thamyongkit, Malick Bachabi, John M. Thompson, Babar Shafiq, Erik A. Hasenboehler

**Affiliations:** 1 Department of Orthopaedic Surgery, The Johns Hopkins University/Johns Hopkins Bayview Medical Center, 4940 Eastern Ave., Baltimore, MD 21224 USA; 2 Chakri Naruebodindra Medical Institute, Faculty of Medicine Ramathibodi Hospital, Mahidol University, 270 Rama VI Road, Ratchatewi, Bangkok, 10400 Thailand

**Keywords:** Cost-control measures, External fixators, Implants, Reprocessing, Trauma surgeons

## Abstract

**Background:**

The increasing financial burden of orthopaedic implants on our health care system has prompted cost-control measures, such as implant reprocessing. The purpose of this study was to describe the current usage by orthopaedic trauma surgeons of reprocessed external fixators (EFs) for treatment of complex fractures.

**Methods:**

A 16-question survey about use and perceptions of reprocessed EFs was distributed to 894 Orthopaedic Trauma Association members between August 2016 and June 2017 using a web-based survey system.

**Results:**

The authors received 243 responses (27%). Thirty-seven percent of respondents reported using reprocessed EFs. Nonprofit hospitals used reprocessed EFs more commonly than did for-profit hospitals (41% vs 15%, *P* = .0004). Eighty-seven percent of respondents believed reprocessing could be cost-effective. The most common reason (32%) for not using reprocessed EFs was coordination/logistics of reprocessing. Concern about litigation was also reported as a main reason for not using (20%) or having recently stopped using (21%) reprocessed EFs.

**Conclusions:**

Many orthopaedic traumatologists are interested in the reprocessing of EF components but few have reprocessing systems in place at their institutions. A major barrier to implementation is concern about litigation, which is likely unwarranted on the basis of Food and Drug Administration approval and a lack of previous litigation. Reprocessing by the original device manufacturers has yielded substantial savings at our institution and is an example of the cost savings that can be expected when implementing an EF reprocessing system.

## Background

External fixation is an important and expensive orthopaedic procedure. The increased use of external fixation during the last decade coincides with the acceptance of staged treatment of complex periarticular fractures and injuries amenable to damage-control approaches [1–4]. However, the financial implications of using costly, single-use implants have driven hospital administrators and surgeons to seek cost-control measures. One solution has been to reprocess parts of these implants for reuse.

Reprocessing can be performed by the original implant manufacturer, a third-party company, or the hospital [5, 6]. Historically, the United States Food and Drug Administration (FDA) did not require reprocessors to comply with the full regulation involved in medical device manufacturing. In response to safety concerns, the FDA began requiring third-party reprocessors and hospitals to adhere to the same regulations as did the original manufacturers [6–8]. This policy has introduced liability concerns for hospitals choosing to reprocess external fixators (EFs) themselves. Similarly, many hospitals have been reluctant to use third-party reprocessing because of the perceived lower standards of inspection and testing compared with those of the original manufacturer. Another concern is the loss of coverage under the original manufacturer’s warranty when using third-party reprocessing. However, in a prospective study, Sung et al. [9] reported no differences in complication rates (e.g., loss of fixation, loosening of the EF) between patients treated with a new versus reprocessed EFs. Notably, the authors reported a cost savings of 25% when using reprocessed EFs.

In response to market demand, orthopaedic device manufacturers have begun their own reprocessing programs for some EF components after receiving FDA 510(k) approval. This clearance for retesting, re-evaluation, and recertification [10] eliminates the hospitals’ responsibility to fulfill FDA regulatory requirements and reduces liability. Another advantage of reprocessing through the original manufacturer is that manufacturers have access to their original specifications and may have heightened interest in the safety of their products [10]. Given the need for hospitals to contain health care costs, financial savings may make reprocessing attractive for hospitals and manufacturing companies.

The purpose of this study was to determine the current practices of orthopaedic trauma surgeons with regard to reprocessed EFs. We hypothesized that there would be heterogeneity in practices between institutions because of a lack of knowledge of the reprocessing options and concerns about safety, liability, FDA approval, and questionable cost savings. We reviewed the legal literature associated with reprocessing external devices to assess historical and projected risks of using reprocessed devices. Finally, we performed a cost analysis of the EF reprocessing program used at our institution.

## Methods

Survey questions were formulated by an orthopaedic resident, orthopaedic trauma fellow, and orthopaedic trauma surgeon. After receiving approval from The Johns Hopkins Medicine institutional review board (#IRB00134437) and the Orthopaedic Trauma Association research committee, we distributed the survey to all Orthopaedic Trauma Association members between August 2016 and June 2017 using a web-based survey system (SurveyMonkey, SurveyMonkey Corp., San Mateo, CA). Privacy was maintained by collecting only de-identified data and using recruitment procedures that did not allow tracing of respondents to answers. Survey participation was voluntary and no incentives were provided.

### Survey design

The survey consisted of 16 questions. Questions 1–7 asked about characteristics of the respondent’s hospital/health care center environment. Questions 8–10 inquired about the respondent’s current practice of using EFs and experience with reprocessed EFs. Questions 11–16 assessed the respondent’s knowledge and perceptions of reprocessed EFs. Respondents were able to provide open-ended answers, which were reported as the multiple choice option that was closest in meaning. If the open-ended answer did not correspond with any multiple choice option, it was treated as a new answer.

### Data analysis

Survey data were analyzed using Excel, version 2016, software (Microsoft Corp., Redmond, WA). Statistical analyses were performed using MedCalc, version 15.0, statistical software (MedCalc Software, Ostend, Belgium). Categorical data were analyzed using Fisher exact tests. *P* values < .05 were considered significant.

### Legal literature review

The literature on reprocessed medical devices was reviewed to address concerns regarding litigation. We performed searches using LexisNexis, Westlaw, and PubMed for legal cases pertaining to all reprocessed devices in orthopaedic and non-orthopaedic surgical specialties without restrictions on publication date. The following medical subject headings and terms were used in the search: “external fixator,” “reprocessing,” “reprocessed,” “reuse,” “reusable,” and “complication.” To confirm search results, the first author and our senior librarian performed the searches independently using the same criteria. The results were compared, and there were no differences between them.

### Reprocessing at our institution

An EF reprocessing system was initiated at our institution in November 2014 with one implant manufacturer (DePuy Synthes, West Chester, PA). FDA-approved components (bar-to-bar clamps, multi-pin clamps, outriggers, and bars) that are eligible for reprocessing and recertification by the vendor were included. To evaluate savings, we calculated the cost of all EF components used during 9 months of reprocessing (January to September 2017). Savings were calculated as the difference between the cost of new components and reprocessed components. We also compared the total number of components used with the number that were recertified through the reprocessing process during the same period to obtain the percentage of total costs saved.

## Results

### Respondents

We sent survey invitations to all 894 Orthopaedic Trauma Association members and received 243 responses (27%). Most respondents worked at nonprofit institutions (84%) and at urban hospitals (56%). Forty-six percent of respondents worked at university-based centers (Table 1).

**Table 1:** Characteristics of the Institutions of 243 Orthopaedic Trauma Association Members Surveyed, August 2016–June 2017

Hospital/Health Care Center Characteristic	No. (%)
Affiliation	
University	111 (46)
Community	109 (45)
Public	23 (9)
Location	
Urban	136 (56)
Suburban	72 (30)
Rural	29 (12)
Other	6 (2)
Type	
Nonprofit	204 (84)
For profit	39 (16)
Other	0 (0)
Size (beds)	
< 200	21 (8.6)
200–800	177 (73)
> 800	44 (18)
Other	1 (0.4)
Number of orthopaedic trauma surgeons	
0–5	214 (88)
6–10	19 (7.8)
> 10	10 (4.1)
Active orthopaedic trauma research program	
Yes	136 (56)
No	107 (44)

### Current practice

Only 37% of respondents reported current use of reprocessed EFs at their institutions. Reasons for not using reprocessed EFs included coordination and logistics of reprocessing (32%), litigation concerns (20%), a gap in knowledge about the process (12%), and doubts about actual cost savings (12%). Recent cessation of reprocessing was reported by 29 respondents. Respondents reported that reprocessing was performed by the original vendor (37%), a third party (34%), or the hospital (12%). Seventeen percent of respondents said they did not know who performed the reprocessing for their institutions (Table 2).

**Table 2:** Current Practices of 243 Orthopaedic Trauma Association Members Regarding the Use of EFs

Practice	No. (%)
Currently use reprocessed EFs	
Yes	90 (37)
No	153 (63)
If not using, considering use of reprocessed EFs	
Yes	95 (62)
No	36 (24)
Undecided	22 (14)
If not using, main reason for not using reprocessed EFs	
Vendor coordination/logistics of reprocessing	49 (32)
Litigation concerns	30 (20)
Gap in knowledge about process	19 (12)
Doubts about actual cost savings	19 (12)
Hospital policy/billing process	15 (9.8)
Concerns about instrumentation failure and limitations	10 (6.5)
Ethical concerns	9 (5.9)
Other/no reason	2 (1.3)
Reasons for recent cessation of using reprocessed EFs^a^	
Vendor coordination/logistics of reprocessing	19 (66)
Litigation concerns	11 (38)
Hospital policy/billing process	8 (28)
Doubts about actual cost savings	7 (24)
Concerns about instrumentation failure and limitations	3 (10)
Ethical concerns	3 (10)
Other/no reason	1 (3.4)
If currently using reprocessed EFs, party performing the reprocessing	
Original manufacturer	33 (37)
Third party	31 (34)
Unsure	15 (17)
Hospital	11 (12)

*EF* external fixator

^a^Twenty-nine respondents reported recent cessation of using reprocessed EFs. Respondents could choose more than one reason

Significantly more nonprofit hospitals used reprocessed EFs (41%) than did for-profit hospitals (15%) (*P* = .0004). The rates of using reprocessed EFs were also significantly different according to hospital affiliation (*P* = .001) (Table 3).

**Table 3:** EF Usage Considerations by Hospital Type, Based on a Survey of 243 OTA Members

EF Usage	Hospital Type, No. (%)			Hospital Affiliation, No. (%)			
	For-Profit (*n* = 39)	Nonprofit (*n* = 204)	*P*	University (*n* = 111)	Community (*n* = 109)	Public (*n* = 23)	*P*
Currently use reprocess EFs							
Yes	6 (15)	84 (41)	0.004	54 (49)	30 (28)	13 (57)	0.001
No	33 (85)	120 (59)		57 (51)	79 (72)	10 (43)	
Believe that EF reprocessing is cost-effective							
Yes	31 (79)	180 (88)	0.320	92 (83)	92 (84)	11 (48)	< 0.001
No	2 (5.1)	5 (2.5)		5 (4.5)	4 (3.7)	1 (4.4)	
Undecided	6 (15)	19 (9.3)		14 (13)	13 (12)	11 (48)	
Considering use of reprocessed EFs							
Yes	18 (55)	77 (64)	0.126	46 (81)	56 (71)	3 (30)	< 0.001
No	12 (36)	24 (20)		5 (8.8)	15 (19)	1 (10)	
Undecided	3 (9.1)	19 (16)		6 (11)	8 (10)	7 (70)	

*EF* external fixator, *OTA* Orthopaedic Trauma Association

### Knowledge and perceptions

When asked what they thought about using reprocessed EFs, most respondents said they believed that reprocessing could be cost-effective (87%) and beneficial for hospital revenue (64%). However, when asked what they perceived to be the most important obstacle to using reprocessed EFs, they cited vendor coordination/logistical challenges of reprocessing (25%), and litigation concerns (20%) (Table 4).

**Table 4:** Perceptions of Reprocessed EFs, Based on Survey of 243 Orthopaedic Trauma Association Members

Perception	No. (%)
Do you believe that EF reprocessing can be cost-effective?	
Yes	211 (87)
No	8 (3.2)
Undecided	24 (10)
Do you believe that EF reprocessing can be beneficial to the hospital in generating revenue?	
Yes	156 (64)
Not beneficial to hospital or patient savings	41 (17)
No, only patient savings	17 (7.0)
Unsure	29 (12)
Most important obstacle to widespread implementation of reprocessing	
Vendor coordination/logistics of reprocessing	61 (25)
Litigation concerns	49 (20)
Concerns with instrumentation failure and limitations	41 (17)
Gap in knowledge about process	39 (16)
Doubts about actual cost savings	32 (13)
Ethical concerns	17 (7.0)
Hospital policy/billing process	2 (0.82)
No concern	2 (0.82)

*EF* external fixator

### Legal concerns

Our search yielded 134 legal cases (83 in PubMed, 37 in LexisNexis, and 14 in Westlaw). Some of these were legal cases related to reuse of medical devices. However, none of the cases was related directly to the reuse of EFs or EF reprocessing.

### Reprocessing at our institution

At our institution, FDA-approved EF components were reprocessed and recertified by the original manufacturer. The overall rate of component recertification (“pass rate”) was 80%. We used reprocessed EFs whenever they were available. However, in every case in which EFs were used, we also needed new components for the EF that are unable to be reprocessed or that failed recertification. The total cost of all EF parts (475) used (bar-to-bar clamps, bars, multi-pin clamps, and outriggers) during the 9-month study period would have been $389,251 if purchased new, whereas the total cost of purchasing the same number of reprocessed EF parts was $97,999. Therefore, the total 9-month cost savings achieved through this system was $291,252.

## Discussion

In the interest of reducing medical costs, one strategy is the use of reprocessed EF devices. Given the novelty of device reprocessing, we sought to assess current orthopaedic traumatologists’ understanding of this system. Our results showed an overall lack of knowledge of the potential value of EF reprocessing. Although most respondents believed that EF reprocessing could be cost-effective (87%) and beneficial to hospital revenue (64%), most respondents (63%) were not using reprocessed components. The most common reasons reported for not reprocessing were vendor coordination (32%) and litigation concerns (20%). A small proportion of respondents (12%) acknowledged their deficient understanding of reprocessing. Increasing surgeon knowledge of reprocessing is a crucial first step in broadening implementation.

A cost analysis of EFs reprocessing at our institution showed a 9-month cost savings of $291,252 (75% savings compared with list price). This would equal nearly $400,000 for 1 year of reprocessing. The result is similar to those of previous reports showing an approximate savings of 25–32% of list prices over time [8–10]. The senior author also collected EF components for charitable donation for 4 years before the implementation of the reprocessing system, with a total component value of more than $900,000, suggesting the potential long-term savings of reprocessing. Considering the financial pressures facing modern healthcare systems, implementation of cost-saving, FDA-approved measures for reprocessed devices such as that for EFs may become crucially important.

Litigation concerns were reported as a main reason for not using (20%) or having recently stopped using (21%) reprocessed EFs. Possible legal issues raised by the hospital related to informed consent of patients about reprocessed instrumentation could complicate the implementation of this process. However, our review of the legal literature found no reports of litigation pertaining to the use of reprocessed EFs. Likewise, the senior author (EAH) has encountered no legal issues at his institution related to the use of reprocessed EFs during the past 2 years. Although the legal process to implement the use of reprocessed EFs was time-consuming to complete, mainly because of the need to establish billing codes and fees for reprocessed components, the reprocessing system has produced substantial savings with no related litigation.

**Fig. 1:**
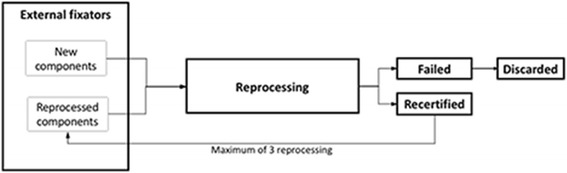
External fixator usage and reprocessing process

At our institution, EF reprocessing was performed by the original manufacturer of the devices according to their guidelines [11]. Used EF components, such as bars, bar-to-bar and pin-to-bar clamps, and outriggers are collected in the operating room and shipped periodically to a centralized reprocessing plant operated by the original manufacturer. Components are then cleaned and tested to ensure mechanical integrity. Each EF component that is recertified under FDA 510(k) regulations is made available for use, and components that fail recertification are discarded. A given component may be reprocessed up to 3 times, as authorized by the vendor and the FDA (Figure 1) [6]. The components that pass inspection and testing are sterilized, packaged, and distributed under a component code that identifies them as “reprocessed” for billing purposes. After the third reuse, all components are discarded automatically. Reprocessing by the original manufacturer standardizes the quality of inspections, guarantees integrity of the implants and simplifies the redistribution to the original buyer using the same logistical paths that are used for newly manufactured devices. Similarly, it simplifies steps for hospitals, who do not need to develop a reprocessing system themselves or work with third-party companies that may not have access to the technical specifications of the equipment or logistical paths that the original manufacturers possess. Of our reprocessed EFs, 80% passed the recertification process during the 6-month study period, which is comparable to the 76% first-pass and 85% second-pass rates reported by Horwitz et al. [10]. We have not observed any mechanical failure or loosening of the reprocessed EF components, suggesting that the reprocessed EFs maintain their mechanical integrity, and that criteria for recertifying components are appropriate.

Legal and ethical considerations for the healthcare system pertain to how reprocessed EFs are billed. The Current Procedural Terminology code (20690) for the application of an EF does not distinguish between the use of a newly manufactured device versus a reprocessed device. However, for appropriate billing, a code different from the one used for new parts is required for the reprocessed components. Implementation of an already FDA-approved reprocessing system at other institutions therefore requires the establishment of unique billing codes for new versus reprocessed components to be used by the institution and the original manufacturer.

Ethical implications for patients regarding billing and familiarity with a reprocessing system must be considered, as well. It would be unethical to charge patients full price for reprocessed components [6]. Similarly, disclosing the use of reprocessed EFs as opposed to newly manufactured devices warrants consideration, because patients may be hesitant to accept the use of reused components [6]. When disclosing the use of reprocessed parts, it may be important to explain to patients that these components are FDA approved and have no known associated risks. We have observed no complications associated with the use of reprocessed components since the implementation of the reprocessing system at our institution. Furthermore, our review of the literature has found no litigation pertaining to the use of reprocessed EFs.

This study has limitations. Because it is based partially on a survey, there are risks of non-responder bias and self-reporting bias that may alter the findings. We did not assess cost saving data from other institutions or vendors; therefore, results may differ in other practices. Further, our savings analysis might not be generalizable to other institutions because final negotiated vendor prices vary among institutions. Finally, our cost analysis may not correlate with those in other countries, where parts are reprocessed by the institutions themselves, or where resources such as water and electricity used for reprocessing and transportation of reprocessed items may be more expensive, thus making single-use newly manufactured components potentially cheaper than reprocessed parts. However, Mahapatra and Rengarajan [5] showed recently that use of reprocessed EF components is safe and financially feasible, even in developing countries.

This study meanwhile has several strengths. Respondents were able to provide open-ended answers to some questions, indicating their actual practices as opposed to being limited by designated responses. All respondents were members of the Orthopaedic Trauma Association who have knowledge of and experience with using EFs. Finally, we obtained a response rate of 27%, which is higher than the typical 10–25% response rates for other Orthopaedic Trauma Association member surveys [12–16].

## Conclusion

Many orthopaedic traumatologists are interested in the reprocessing of EF components but few currently have reprocessing systems in place at their institutions. A major barrier to implementation is concern about litigation, which is likely unwarranted on the basis of FDA approval and a lack of previous litigation. Reprocessing by the original device manufacturers has yielded substantial savings at our institution and is an example of the cost savings that can be expected when implementing an EF reprocessing system.

## Abbreviations

EF: External fixators
FDA: Food and Drug Administration
